# Induced Resistance in *Solanum lycopersicum* by Algal Elicitor Extracted from *Sargassum fusiforme*

**DOI:** 10.1155/2015/870520

**Published:** 2015-02-24

**Authors:** Layth Sbaihat, Keiko Takeyama, Takeharu Koga, Daigo Takemoto, Kazuhito Kawakita

**Affiliations:** ^1^Plant Pathology Laboratory, Graduate School of Bioagricultural Sciences, Nagoya University, Chikusa-ku, Nagoya 464-8601, Japan; ^2^Plant Disease and Insect Control Group, Aichi Agricultural Research Center, Yazakosagamine, Nagakute 480-1103, Japan; ^3^Research and Development Group, Ohta Oil Mill Co., Fukuoka-cho, Okazaki 444-0825, Japan

## Abstract

Tomato (*Solanum lycopersicum*) production relies heavily on the use of chemical pesticides, which is undesired by health- and environment-concerned consumers. Environment-friendly methods of controlling tomato diseases include agroecological practices, organic fungicides, and biological control. Plants' resistance against pathogens is induced by applying agents called elicitors to the plants and would lead to disease prevention or reduced severity. We investigated the ability of a novel elicitor extracted from the brown sea algae (*Sargassum fusiforme*) to elicit induced resistance in tomato. The studied elicitor induced hypersensitive cell death and O_2_^−^ production in tomato tissues. It significantly reduced severities of late blight, grey mold, and powdery mildew of tomato. Taken together, our novel elicitor has not shown any direct antifungal activity against the studied pathogens, concluding that it is an elicitor of induced resistance.

## 1. Introduction

Plant pathogens are the largest competitor of agricultural crops and severely reduce the crop production in the range of 25–50% [1, 2]. To protect agricultural crops, enormous amounts of synthetic fungicides are used over the world. The total value of world's agrochemical market was between US$31 and 35 billion [3]. However, the excessive use of synthetic fungicides in the croplands, urban environment, and water bodies has resulted in an increased risk of fungicides resistance, enhanced pathogen resurgence and development of resistance/cross-resistance, toxicological implications to human and environmental health, and increased pollution [4–6].

Treatment of plants with various agents, including cell wall fragments, plant extracts, and synthetic chemicals, can induce resistance to subsequent pathogen attack both locally and systemically [7]. Agents inducing plant's resistance are called elicitors. Elicitors are compounds that induce accumulation of antimicrobial phytoalexins and any type of defense response [8]. Elicitors have been isolated from bacteria, fungi, oomycetes [9], sea algae [10], and plants or even chemically synthesized [7] and can be proteins, peptides, fatty acids, glycoproteins, lipids, oligosaccharides, and polysaccharides [9–11]. Complete pathogen control is rarely proven to occur by elicitor-induced resistance but often results in reducing lesion size and/or number [12].

Marine algae extracts are used as spray fertilizers on a number of crops, with a wide range of beneficial effects such as improved yield, increased resistance to biotic or abiotic stress, and control of flowering and of fruit maturation. Actually, marine algae represent an abundant and naturally occurring source of potential elicitors. Due to the activity of algal extracts as plant protectants, it was proposed that they might act as elicitors of plant defense responses. An extract of the brown algae* Laminaria digitata* was shown to induce several defense responses in tobacco cell suspension cultures [13]. It also induces grapevine resistance reactions leading to protection against* Botrytis cinerea* and* Plasmopara viticola* [14]. Cell walls components of marine brown algae induce the formation of antifungal compounds in alfalfa cotyledons [15]. They stimulate several resistance reactions in tobacco suspended cells and consistently induce both local resistance and systemic resistance to tobacco mosaic virus (TMV) [16]. Elicitors extracted from the cell walls of red algae were shown to elicit laminarinase (1,3 (beta)-D-glucanase) and phenylalanine ammonia lyase (PAL) enzymes involved in plant defense reactions [17] and played as potent elicitorsof defense in tobacco plants [18].* Medicago truncatula* plants infiltrated or sprayed with an extract from green algae,* Ulva* spp., were protected against the fungal pathogen* Colletotrichum trifolii,* following changes in the expression of a large number of plant defense genes [19].

Tomato (*Solanum lycopersicum*) is one of the most widely grown vegetable crops in the world. The production in the world is about 160 million tons fresh fruit from 4.7 million ha [20]. Tomato production is severely constrained by diseases and pests. Growers rely heavily on chemical fungicides and pesticides to protect their crops [21]. For example, farmers in southern India spray chemicals more than 50 times during a cropping season [22]. The intensive use of fungicides also leads to increased cost of agricultural production. Researchers increasingly tend to investigate and develop elicitors of plant defense that can substitute the use of fungicides for high fungicides demanding crops like tomato. Several elicitors were reported to induce resistance reactions in tomatoes; however, among them only salicylic acid (SA) and chitosan induced the tomato's resistance to pathogens [23]. In this study, we analyze the ability of a crude water extract of the brown sea algae (*Sargassum fusiforme*) to induce resistance reactions, that is, reactive oxygen species (ROS) production and hypersensitive reaction (HR) in* S. lycopersicum* (cv. House Momotaro) plants. We also studied the ability of the extract to induce the plants' resistance against major fungal diseases.

## 2. Materials and Methods

### 2.1. Biological Materials and Growth Conditions

Tomato plants (*S. lycopersicum *cv. House Momotaro) were germinated from seeds (Takii seed co., Kyoto, Japan) at 25–28°C and then grown at 20–23°C under a photoperiod of 16 h light/8 h dark period in environmentally controlled growth cabinets. The pathogenic isolate of the hemibiotrophic oomycete* Phytophthora infestans* (Mont.) De Bary (race 1.2.3.4) was used in this research. Zoosporangia of* P. infestans* were extracted and zoospore production was induced. Twenty mL water was added to the surface of 7–10 days old subcultures of* P. infestans*, which were then rubbed with a cotton swab to release zoosporangia. Zoosporangia suspensions were then incubated at 10°C for 3 h for producing zoospores. Conidia of* Botrytis cinerea* were produced by subculturing strain (B-4) on PDA media for 6–8 days, at 25°C and under exposure to near-ultraviolet light (NEC FL20SBL-B). Conidia were harvested in water by a cotton swab. Tomato plants infected with the obligate biotroph* Oidium spp*. were kept at 23–25°C under a photoperiod of 16 h light/8 h dark period in environmentally controlled growth cabinets.

### 2.2. Inoculation

For plant pathogen interaction tests, leaflets of tomato plants were inoculated with 0.5 mL aliquots of* P. infestans* zoospore (10^5^ zoospores/mL) and covered with lens papers; the inoculated plants were kept at high humidity and 20°C for 1 day and were then moved into 23°C growth cabinet. Tomato leaves were inoculated with 50 *μ*L drops of* B. cinerea *spores (10^6^ spore/mL) and were kept in 23°C growth cabinets. Tomato plants were mixed with plants infected with* Oidium spp.* and the positions of the plants were changed randomly every two days to insure uniformity of exposure to the airborne pathogen. The inoculated plants were observed on daily basis for monitoring disease severity on plants leaves.

### 2.3. Elicitor Extraction and Preparation

Brown sea algae (*S. fusiforme*) were steamed at a temperature of 120°C and a pressure of 2.0 kg/cm^2^ for 60 minutes. The steam was trapped and cooled. The obtained solution was used as sea algal product (AP).

### 2.4. Measurement of ROS Production

L-012 (Wako, Osaka, Japan) is a luminol derivative that is highly sensitive to superoxide anion (O_2_^−^). To detect the O_2_^−^ production in tomato leaves, 0.5 mM L-012 in 10 mM MOPS-KOH (pH 7.4) was infiltrated to the intercellular space of leaves via a needleless syringe. A photon image processor equipped with a sensitive CCD camera was used to continuously monitor chemiluminescence in a dark chamber at 20°C (AquaCosmos 2.5; Hamamatsu Photonics, Shizuoka, Japan) and was quantified using the U7501 program (Hamamatsu Photonics).

### 2.5. Detection of Hypersensitive-Like Cell Death

To detect the hypersensitive reaction- (HR-) like cell death in plants, elicitors were infiltrated using a needleless syringe through the abaxial surface of the leaves. To visualize plant cell death,* S. lycopersicum *leaves were stained with lactophenol trypan blue [24] with minor modification. Briefly, infected leaves were cleared in methanol overnight, and then the cleared tissues were boiled for 2 min in lactophenol trypan blue stain (10 mL of H_2_O, 10 mL of lactic acid, 10 mL of glycerol, 10 g of phenol, and 10 mg of trypan blue). After the leaves were allowed to cool at room temperature for 1 h, the stain was replaced with a fixing solution (1 g/mL chloral hydrate). Stained leaves were visualized using a microscope (Olympus BX51, Tokyo, Japan).

### 2.6. Antifungal Assay

The antifungal activity of algal elicitor was studied using “agar disk diffusion method,” where filter disk infiltrated with AP, H_2_O, or Hygromycin B (150 *μ*g/mL) was introduced into ray and PDA media subcultured with* P. infestans* and* B. cinerea* isolates, respectively. Growth inhibition effect was visualized and the plates were photographed.

### 2.7. Statistical Analysis

Data were analyzed separately for each experiment using the “SPSS 12.0” program, means were compared using student's *t*-test, and differences were compared at *P* < 0.05 or *P* < 0.01.

## 3. Results and Discussion

### 3.1. Results

#### 3.1.1. Algal Extract Treatment Reduced Powdery Mildew Disease in Tomato Plants

**Figure 1:**
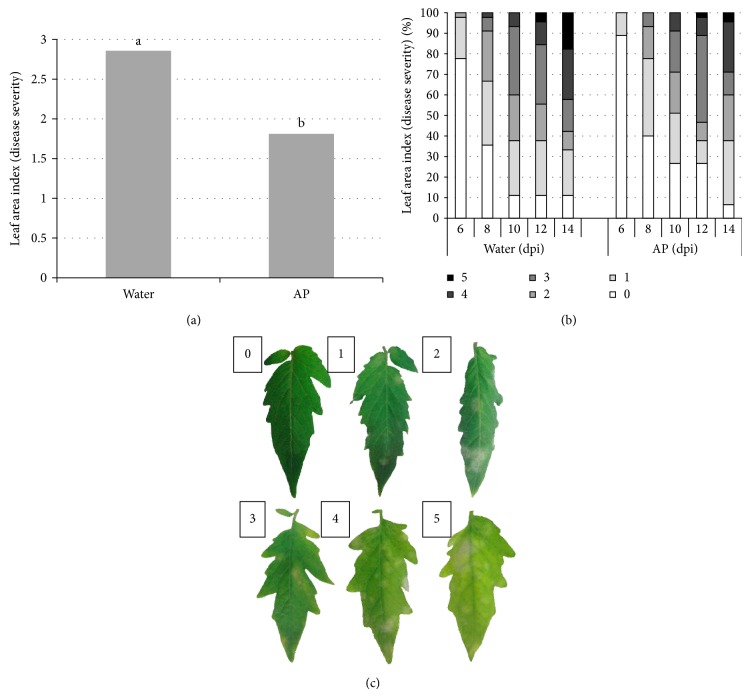
Plant pathogen (*S. lycopersicum* cv. House Momotaro—*Oidium spp.*) interaction in the presence of algal product (AP) elicitor. Tomato (HM) plants were spray-washed with AP (1%) or H_2_O and introduced into a growth chamber containing* Oidium spp. *infected plants 1 day later, and disease development was assessed daily from the 6th until the 14th day after the introduction. (a) Disease severity on* S. lycopersicum* leaves 10 days after disease introduction. Different letters indicate significant differences at (*P* < 0.05). (b) Percentage of infected leaves and disease severity of the AP- and H_2_O-treated plants from 6th until 14th day after pathogen introduction. Disease severity was quantified in reference to (c). (c) Development of* Oidium spp. *on* S. lycopersicum* leaves was marked from 0 (if the leaf was not infected) to 5 (if the leaf was fully infected) and this reference was made for this purpose accordingly. Three plants were used in each experiment. Results are the average of three independent experiments.

Foliar applications of AP onto* S. lycopersicum* (cv. House Momotaro) plants showed reduction of the powdery mildew disease incidence and severity on the plants. An experiment was designed where tomato plants were thoroughly sprayed with AP or water and introduced into a growth chamber containing tomato plants infected with* Oidium spp.* and monitored for the infection and development of the airborne pathogen on the leaves (Figure 1). Leaves subjected to the study were marked according to disease development from 0 (for completely healthy leaf) to 5 (for the thoroughly infected leaf) (Figure 1(c)). Results show significant reduction of over 37% of disease severity on the AP treated plants monitored 9 days after disease introduction (Figure 1(a)).  Figure 1(b) shows percentages of infected leaves and disease severity from the 6th day until the 14th day after disease introduction. Actually, the powdery mildew pathogen could infect fewer* S. lycopersicum *leaves and at less severity throughout the whole testing period.

#### 3.1.2. Algal Extract Treatment Reduced Late Blight and Gray Mold Diseases in Tomato Plants

**Figure 2:**
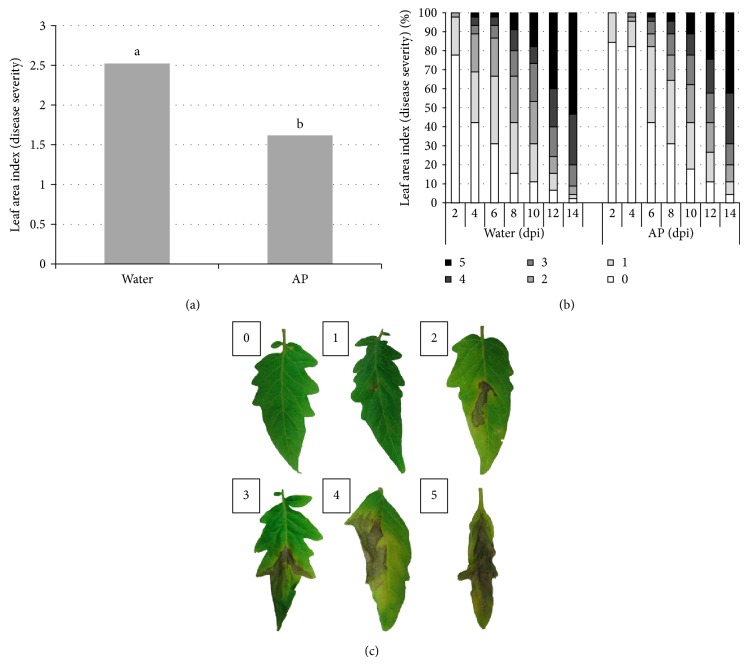
Plant pathogen (*S. lycopersicum* cv. House Momotaro—*P. infestans*) interaction in the presence of algal product (AP) elicitor. Tomato (HM) plants were thoroughly sprayed with AP (1%) or H_2_O and inoculated with 1 mL of* P. infestans* (race 1.2.3.4) spores (10^6^ spore/mL) 1 day later and kept in a humid chamber for another 24 h, and disease development was assessed daily from the 2nd day until the 14th day after the introduction. (a) Disease severity on* S. lycopersicum* leaves 7 dpi. Different letters indicate significant differences at (*P* < 0.05). (b) Percentage of infected leaves and disease severity of the AP- and H_2_O-treated plants from 2nd dpi until 14th dpi. Disease severity was quantified in reference to (c). (c) Development of* P. infestans *on* S. lycopersicum* leaves was marked from 0 (if the leaf was not infected) to 5 (if the leaf was fully infected) and this reference was made for this purpose accordingly. Three plants were used in each experiment. Results are the average of three repeated experiments.

Experiments were designed to assess the effect of AP against the late blight hemibiotrophic pathogen (*P. infestans*) and the gray mold necrotrophic pathogen (*B. cinerea*). Tomato plants were thoroughly sprayed with AP or water and inoculated with* P. infestans* spores 24 h later and monitored for late blight disease development on the leaves from the 2nd until the 14th dpi (Figure 2). Leaves subject of the study were marked according to disease development from 0 (for completely healthy leaf) to 5 (for the thoroughly infected leaf) (Figure 2(c)). Similar to the results obtained in regard topowdery mildew, late blight severity was significantly reduced (around 36%) on AP-treated plants assessed 7 dpi (Figure 2(a)). Also, the number of infected leaves and disease severity of AP-treated plants were lower than those of the water-treated plants. AP application offered the plants disease protection for the whole period of the experiment from the 2nd until the 14th dpi (Figure 2(b)).

**Figure 3:**
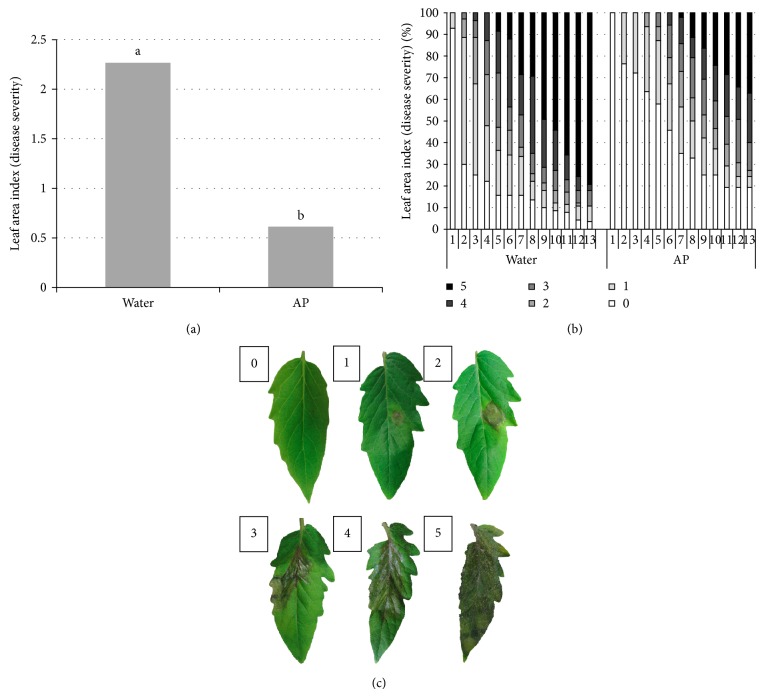
Plant pathogen (*S. lycopersicum* cv. House Momotaro—*B. cinerea*) interaction in the presence of algal product (AP) elicitor. Tomato (HM) plants were spray-washed with AP (1%) or H_2_O and inoculated with 15 *μ*L of* B. cinerea *spores (10^5^ spore/mL) 1 day later, and disease development was evaluated daily from the first dpi until the 13th dpi. (a) Average disease severity evaluated 5 dpi. Different letters indicate significant differences at (*P* < 0.01). (b) Percentages of infected* S. lycopersicum* leaves and disease symptom severities of the AP- and H_2_O-treated plants from 1st until the 14th dpi. Disease severity was quantified in reference to (c). (c) Development of* B. cinerea* on* S. lycopersicum* leaves was marked from 0 (if the leaf was not infected) to 5 (if the leaf was fully infected) and this reference was made for this purpose accordingly. Three plants were used in each experiment. Results are the average of three repeated experiments.

AP preceding application protected tomato plants against gray mold disease. When tomato plants were sprayed with AP, they showed lower susceptibility to the necrotrophic* B. cinerea* inoculated subsequently (Figure 3). Disease severity on the AP-sprayed* S. lycopersicum *plants was reduced by 80% compared to the control observed 5 dpi (Figure 3(a)). In addition, notable reduction in the number of infected leaves and disease severity were noticed throughout the 13 days following* B. cinerea* spores inoculation (Figure 3(b)).

#### 3.1.3. Algal Extract Has No Direct Effect against Late Blight and Gray Mold Pathogens

**Figure 4:**
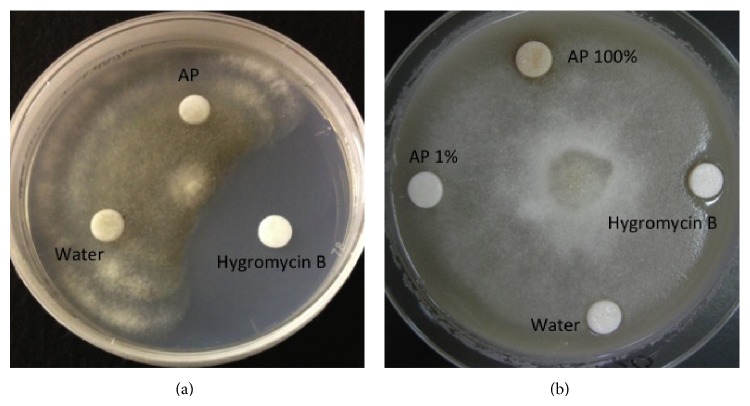
Algal extract showed no antifungal activity against* P. infestans* or* B. cinerea*. (a) Disks infiltrated with 20 *μ*L of AP (100%), AP (1%), and Hygromycin B (150 *μ*g/mL) as a positive control or H_2_O as a negative control. Photograph was taken 5 days after inoculation. (b) Disks infiltrated with 20 *μ*L of AP (100%), Hygromycin B (150 *μ*g/mL) as a positive control or H_2_O as a negative control. Photograph was taken 7 days after inoculation. Experiment were repeated three times and showed similar results.

To investigate whether the noticed disease protection was caused by the direct antifungal effect of AP, we examined the antifungal activity of AP against the studied pathogens. AP did not show any growth inhibition of* P. infestans* and* B. cinerea* when applied through desk diffusion tests into cultures on ray grass and PDA media, respectively (Figure 4).

#### 3.1.4. Algal Extract Treatment Induced Resistance Reactions in Tomato Plants

**Figure 5:**
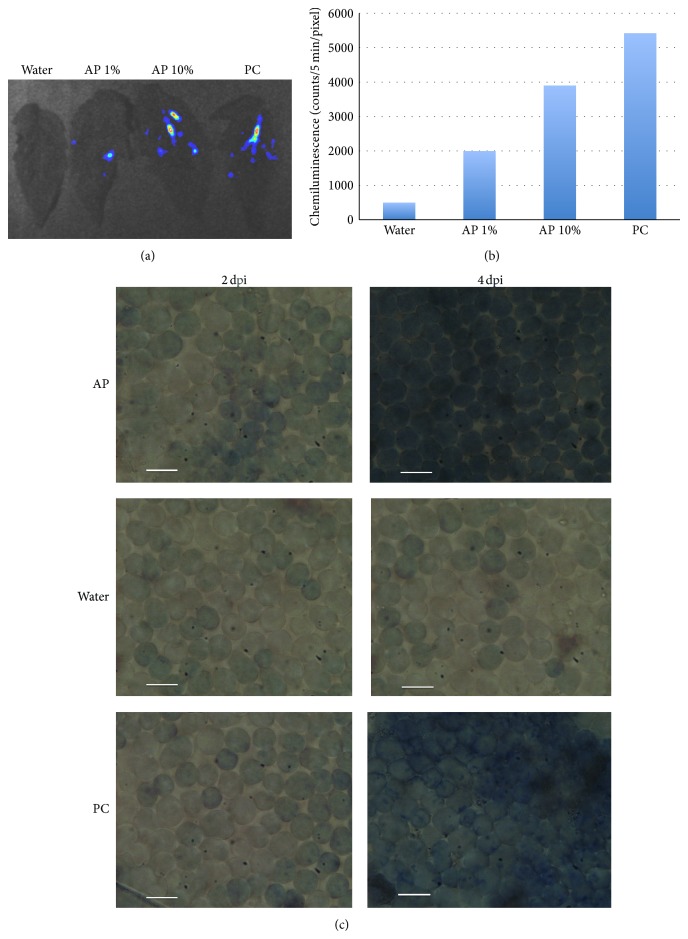
AP induced resistance reaction in tomato plants. (a)* Solanum lycopersicum* plants were sprayed with AP 1%, AP 10%, and H_2_O (as a negative control) or hyphal wall components (HWC) of* P. infestans* (as a positive control), and 90 min later the luminol derivative L-012 (0.5 mM L-012 in 10 mM MOPS-KOH (pH 7.4)) was infiltrated to the abaxial surface of the leaves using a needleless syringe. Chemiluminescence was monitored using a photon image processor equipped with a sensitive CCD camera in a dark chamber at 20°C. Representative graphs were taken 90 mpt. (b) Data were quantified using the U7501 program. Shown data are the average of three repeated experiments. Bars with different letters are significantly different at (*P* < 0.05). (c)* S. lycopersicum* leaves were infiltrated through the abaxial surface using a needleless syringe with AP 10% and H_2_O (as a negative control) or HWC (0.5 mg/mL) (as a positive control) and incubated for 2 or 4 days and then visualized for HR-like cell death using trypan blue staining method as described in Section 2.

Since AP reduced disease development but showed no direct effect against the pathogens, it raised the possibility that AP is an elicitor inducing the plants' resistance against pathogens. Tomato plants were sprayed with AP at two concentrations, water as a negative control and hyphal wall components (HWC) of* P. infestans* reported to induce resistance reactions in plants that belong to the Salicaceae family [25] as a positive control, and were examined for O_2_^−^ accumulation 90 min after treatment (mpt). O_2_^−^ production was induced in tomato plants as a result of AP application at the concentrations of 1% and 10% (Figure 5(a)). Moreover, O_2_^−^ producing activity of AP-treated tomato leaves appeared dose-dependent when quantified by photon counting using the “U7501 program” (Figure 5(b)).

The studied AP has also shown hypersensitive-like cell death induction in tomato tissues. Tomato leaves were infiltrated with AP and HR-like cell death was visualized using trypan blue staining 2 and 4 dpt. HR-like cell death was noticed 2 days after treatment (dpt), but a massive cell death was noticed 4 dpt (Figure 5(c)).

### 3.2. Discussion

Tomato (*Solanum lycopersicum*) is a world important vegetable crop, which is severely constrained by diseases and pests. Uncovering elicitors that may replace the genetically modified plants and the heavy use of agrochemicals in tomato agriculture grasped researchers' attention. Several elicitors were reported to induce resistance reactions in tomato; for instance, oligogalacturonic acid (OGA) was reported to inhibit light-induced stomatal opening and to accelerate stomatal closing of tomato plants, and it also induced the induction of reactive oxygen species production in guard cells [26]. Oligogalacturonides of fungal and bacterial origin induced protein inhibitors synthesis and defense genes activation [27]. Hypersensitive response (HR) of tomato was activated as a result of application of Avr gene products (AVR4 and AVR9) from* Cladosporium fulvum*, viral coat protein hairpin from TMV, and sphinganine analogue mycotoxins from* Fusarium moniliforme* [28, 29]. In addition, sphinganine analogue mycotoxins also activated defense related genes in tomato plants [29]. Chitosan inhibited light-induced* * stomatal opening, increased levels of catalase and peroxidase enzymes activities, and elevated tomato's resistance against* Fusarium oxysporum* and* Phytophthora capsici* [26, 30, 31]. Exogenous applications of salicylic acid onto tomato plants upregulated the transcription of PR1 and BGL2 genes (marker genes of SA pathway) and increased the endogenous level of H_2_O_2_ [32]. Salicylic acid increased the levels of catalase and peroxidase enzymes activity when applied at tomato fruits [31].

It was proposed that extracts of sea algae might act as elicitors of plant defense responses due to their activity as plant protectants. Actually, marine algae represent an abundant, naturally occurring source of potential elicitors. The *β*-1,3-glucan laminarin derived from the brown algae* Laminaria digitata* was shown to both induce several defense responses in tobacco cell suspension cultures [13] and induce grapevine resistance reactions leading to protection against* Botrytis cinerea* and* Plasmopara viticola* [14]. Sulfated fucans, which are common structural components of the cell walls of marine brown algae, induce the formation of antifungal compounds in alfalfa cotyledons [15] and several resistance reactions in tobacco suspended cells and consistently stimulate both local resistance and systemic resistance to tobacco mosaic virus (TMV) [16]. The sulfated linear galactans, Carrageenans, which are found in the cell walls of many red algae, were shown to elicit laminarinase (1,3 *β*-D-glucanase) and phenylalanine ammonia lyase (PAL) enzymes involved in plant defense reactions [17] and played as potent elicitors of defense in tobacco plants [18].* Medicago truncatula *plants infiltrated or sprayed with an extract from green algae,* Ulva *spp., were protected against the fungal pathogen* Colletotrichum trifolii, *and changes in the expression of a large number of plant defense genes were also reported [19].

Powdery mildew caused by* Oidium spp.* has recently been recognized as a worldwide emerging pathogen on tomato [33]. There are two known tomato powdery mildew species in the* Oidium *genus,* O. lycopersici *occurring in Australia and* O. neolycopersici *in the rest of the world [34, 35]. Commercial tomato cultivars (including House Momotaro) available in the Japanese market in 2003 were found susceptible to the powdery mildew pathogen [36]. We sprayed healthy tomato plants with AP or water and after that, the plants were challenged by* Oidium spp.* Interestingly, AP sprayed plants remain healthier than water sprayed ones throughout two weeks of experimentation. Protection of the plants by AP reached a maximum level 9 days after pathogen introduction with 37% less infected leaves and less disease severity (Figure 1). In a similar experiment, severity of late blight—caused by the hemibiotrophic oomycete* P. infestans*—was similarly reduced by 36% 6 days after* P. infestans* inoculation. AP-treated plants were relatively protected and disease severity was limited throughout the time course of the experiment (Figure 2). Moreover, when tomato plants were treated with the AP and inoculated subsequently with the necrotrophic* B. cinerea*, disease symptoms appeared five times greater in the water-treated plants compared to the AP-treated ones.

We tested the direct antifungal activity of AP against* P. infestans* and* B. cinerea* using disk diffusion method. Our results show no growth inhibition activity of AP against the studied pathogens (Figure 4). We studied the ability of AP to induce two resistance reactions of tomato plants. When tomato leaves were infiltrated with AP, a fast and significant induction of O_2_^−^ occurred (Figures 5(a) and 5(b)). O_2_^−^ is of the early plants resistance reactions induced following pathogen infection or elicitor application, which is involved in direct pathogen control and the pathways of other resistance reactions [37]. Moreover, trypan blue staining experiments show hypersensitive-like cell death clearly noticed in tomato tissues 4 after AP applications (Figure 5).

Resistance reactions were induced in tomato plants as a result of AP applications. AP possesses no antifungal effect against the studied fungal pathogens. The studied extract of* S. fusiforme* shows significant protection of tomato plants against three fungal pathogens of tomato. Elicitor-induced resistance rarely leads to complete pathogen control, but reducing lesion size and/or number instead [12]. Taken together, we conclude that AP-protection of tomato plants was achieved through induced resistance of the plants. Due to the abundance of the source of the extract and its simple preparation, AP can be developed as a safe and environment-friendly elicitor of biological origin, which can participate in tomato cultures protection against fungal diseases.
